# DPPPRED-IV: An Ensembled QSAR-Based Web Server for the Prediction of Dipeptidyl Peptidase 4 Inhibitors

**DOI:** 10.3390/ijms26125579

**Published:** 2025-06-11

**Authors:** Laureano E. Carpio, Marta Olivares, Rita Ortega-Vallbona, Eva Serrano-Candelas, Yolanda Sanz, Rafael Gozalbes

**Affiliations:** 1 MolDrug AI Systems SL, Parque Tecnológico de Valencia, 46980 Valencia, Spain; lcarpio@protoqsar.com; 2 ProtoQSAR SL, Parque Tecnológico de Valencia, 46980 Valencia, Spain; rortega@protoqsar.com (R.O.-V.); eserrano@protoqsar.com (E.S.-C.); 3 Microbiome Innovation in Nutrition & Health Research Unit, Institute of Agrochemistry and Food Technology, Spanish National Research Council (IATA-CSIC), 46980 Valencia, Spain; m.olivares@iata.csic.es (M.O.); yolsanz@iata.csic.es (Y.S.)

**Keywords:** quantitative structure–activity relationships, type 2 diabetes mellitus, dipeptidyl peptidase 4 (DPP4) inhibitors

## Abstract

Type 2 diabetes mellitus (T2DM) is a complex and prevalent metabolic disorder, and dipeptidyl peptidase 4 (DPP4) inhibitors have proven effective, yet the identification of novel inhibitors remains challenging due to the vastness of chemical space. In this study, we developed DPPPRED-IV, a web-based ensembled system integrating both binary classification and continuous regression Quantitative Structure Activity Relationships (QSAR) models to predict human DPP4 inhibitory activity. A curated dataset of 4 676 ChEMBL compounds was subjected to genetic algorithm descriptor selection and multiple machine learning algorithms; classification models were combined via a soft voting ensemble, while regression models estimated IC_50_ values. All models underwent external 10-fold cross-validation and applicability domain analysis. The final models were integrated into a user-friendly web server, allowing predictions from SMILES inputs. Experimental testing of 29 MolPort compounds at 1.5 µM confirmed that 14 predicted actives exhibited significant inhibition, supporting the tool’s performance in early-stage screening. DPPPRED IV is freely available within the ChemoPredictionSuite and offers a resource to accelerate decision making, reduce costs and minimize animal use in T2DM drug discovery.

## 1. Introduction

The global rise in diabetes mellitus and its complications poses a critical public health challenge. In 2024, the International Diabetes Federation reported that approximately 589 million adults aged 20–79 were living with diabetes worldwide, which equates to 1 in 9 adults. This number is projected to rise to about 853 million by 2050, representing 1 in 8 adults [1]. However, these projections may underestimate the true burden of diabetes, especially in regions undergoing rapid epidemiological transitions [2]. The current diabetes epidemic is driven by a combination of factors, including aging populations, urbanization, economic changes, sedentary lifestyles, and unhealthy diets.

Diabetes mellitus is a multifaceted metabolic disorder defined by chronic hyperglycemia, resulting from insufficient insulin production, insulin resistance, or both, and accompanied by disturbances in lipid, protein, and mineral metabolism [3]. The most common forms include type 1 diabetes mellitus (T1DM), type 2 diabetes mellitus (T2DM), and gestational diabetes, as well as less common types caused by infections, drugs, or genetic factors [4].

**Figure 1:**
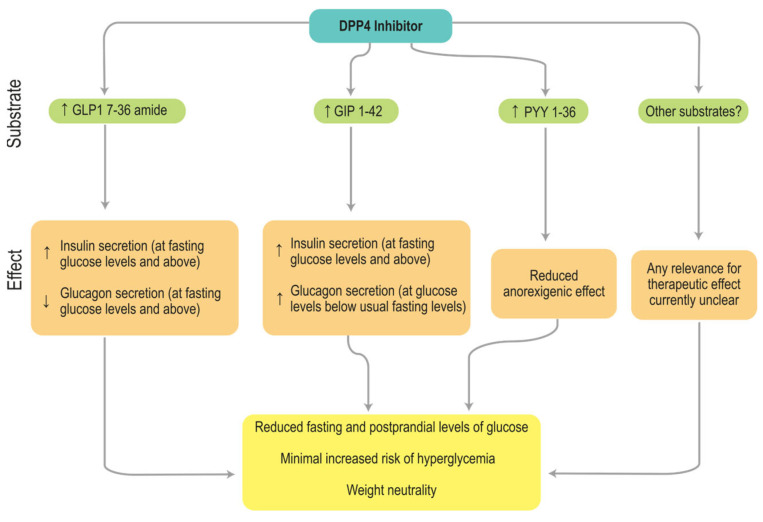
Mechanism of action of DPP4is.

T2DM, which represents over 90% of all diabetes cases [5], emerges from a complex interplay [3] of genetic predisposition and environmental triggers [6]. The disease begins with insulin resistance in key tissues such as the liver, muscle, and adipose tissue, followed by progressive β-cell dysfunction leading to reduced insulin secretion [7]. These two defects, insulin resistance and β-cell failure, are recognized as early pathogenic events in T2DM [8]. Early compensatory hyperinsulinemia gives way to worsening hyperglycemia as β-cell capacity declines, often in the context of overweight or obesity, and T2DM is frequently associated with dyslipidemia, hypertension, and pro-thrombotic states [9,10,11]. Building on the complex interplay between metabolic dysfunction and T2DM, attention has increasingly turned to molecular regulators of glucose homeostasis. One such key player is the dipeptidyl peptidase-4 (DPP4). DPP4, also known as CD26, is a multifunctional serine protease widely expressed as both a membrane-bound and soluble enzyme in tissues, including the intestine, kidney, pancreas, liver, and immune cells [12,13,14,15,16,17]. In addition to its catalytic role in cleaving regulatory peptides, DPP4 modulates cell signaling and immune responses, for example, acting as a co-stimulatory molecule in T-cell activation, underscoring its physiological importance beyond metabolism [13,15,16,18]. The discovery that incretin hormones such as GLP-1 and GIP are physiological substrates rapidly focused attention on DPP4 as a therapeutic target: by preventing GLP-1 degradation, DPP4 inhibition enhances glucose-dependent insulin secretion and suppresses glucagon release while avoiding weight gain typically seen with other agents (Figure 1) [18,19,20,21,22,23,24,25,26,27,28].

Early lead compounds were based on dipeptide-mimetic scaffolds, and subsequent SAR studies yielded first-generation DPP4 inhibitors such as vildagliptin and saxagliptin [29,30,31]. Structural biology breakthroughs, including the resolution of the human DPP4 crystal structure and characterization of its S9B subfamily homology, enabled the design of second-generation agents (sitagliptin, alogliptin, linagliptin) with improved potency, selectivity, and pharmacokinetics [32,33,34,35]. Today’s DPP4 inhibitors comprise a chemically diverse group of molecules with favorable safety and tolerability profiles compared to both older antidiabetic therapies and newer classes, such as SGLT2 inhibitors (Table 1) [36,37,38,39,40].

While generally well-tolerated, DPP4is are not devoid of adverse effects, which may be categorized as class-wide or molecule-specific. Class effects stem from the enzyme’s involvement in immune function and the cleavage of a wide range of bioactive peptides [13,41,42]. These concerns prompted safety evaluations regarding potential immune suppression or cytokine imbalances. However, since the immune-modulating functions of DPP4 (e.g., T-cell co-stimulation) are largely non-enzymatic, clinical evidence indicates that DPP4is does not compromise innate or adaptive immune responses, even in immunocompromised individuals [18,28]. Regarding molecule-specific effects, most reported adverse events, such as mild gastrointestinal symptoms, are rare and less frequent than those seen with GLP-1 receptor agonists like liraglutide [43]. Nonetheless, due to the clinical relevance of the pathology, it remains crucial to maintain efforts toward the identification and optimization of novel DPP4is. Nonetheless, the continuing need for novel DPP4 inhibitors with enhanced efficacy and safety profiles motivates the use of computational chemoinformatics methods.

Considering the therapeutic importance of DPP4 inhibition for the treatment of T2DM and the chemical diversity among available inhibitors, computational tools have become essential for understanding the relationship between the structure and the activity and guiding the development of new compounds. Chemoinformatics, a field at the intersection of chemistry and information technology, offers a framework for managing and analyzing large volumes of chemical data [44,45]. By applying statistical and machine learning (ML) methods to these data, chemoinformatics enables the identification of patterns linking molecular structure with physicochemical, biological, or pharmacological properties. Among the different strategies employed, Quantitative Structure–Activity Relationship (QSAR) modeling stands out for its ability to mathematically relate chemical descriptors to specific molecular activities, making it a powerful predictive tool in drug discovery.

QSAR modeling, rooted in the early hypothesis by Crum Brown that chemical structure determines biological activity [46], is particularly valuable in the rational design of bioactive molecules. In the context of DPP4 inhibition and diabetes research, QSAR models provide insight into the structural requirements for inhibitory potency and selectivity, supporting the discovery of novel antidiabetic agents [47,48,49,50,51].

Building on these concepts, this study introduces DPPPRED-IV, a web-based QSAR platform that integrates binary classification and continuous regression models to predict human DPP4 inhibitory activity. The tool delivers both binary activity predictions and IC_50_ estimates, thereby enabling evidence-based prioritization of candidate molecules for experimental follow-up. By streamlining hit selection, reducing screening time and costs, and minimizing animal use in accordance with the three Rs of replacement, reduction, and refinement, DPPPRED-IV helps to accelerate the early-stage discovery of novel therapeutics for type 2 diabetes.

## 2. Results

### 2.1. Feature Selection, Classification Model Development, and Optimization for Predicting DPP4 Inhibition

**Figure 2:**
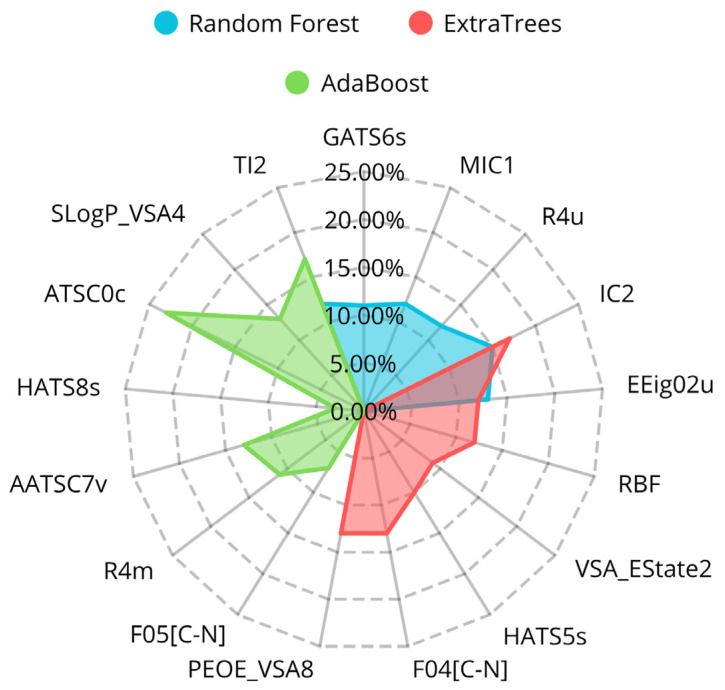
Radial plot of the feature importances of the descriptors for each of the different classification models.

Following the data collection and curation process indicated in Materials and Methods Section 4.1, a final classification dataset was established, consisting of 3929 molecules, from which 1501 are active and 2428 inactive. Using a genetic algorithm (GA), we generated a population of distinct classification models, each developed using different ML algorithms: AdaBoost, Extra Trees, and Random Forest. Models were ranked based on their F1 score, and the best-performing models were selected for further analysis. Based on this, three models were selected, each one with a different algorithm. The molecular descriptors used in these models, along with their feature importance, are shown in Figure 2 and listed with their descriptions in Table 2.

The hyperparameters of these models were optimized, and the final models were used to build ensemble classifiers using both hard voting (HV) and soft voting (SV) strategies. In the SV scheme, weights were systematically tested across a range from 0 to 2.0 to maximize prediction performance. The optimal configuration assigned different weights for the Random Forest, the Extra Trees, and the AdaBoost, with final weights of 0.1, 0.7, and 0.2, respectively.

Model performance was assessed using a 10-fold external cross-validation, applying a range of standard classification metrics to evaluate both individual models and ensemble approaches. The results of this evaluation are summarized in Table 3, demonstrating the improved performance of the ensemble strategies over individual models.

Despite the strong individual performance of the Extra Trees model, evaluation of the validation set (VS) metrics revealed that the SV ensemble achieved compared accuracy to any single model or the HV counterpart. Most notably, the SV ensemble achieved an F1-score of 0.73, along with high precision, satisfactory recall, and strong Matthews Correlation Coefficient (MCC) values.

Although Extra Trees slightly outperformed the ensemble in some individual metrics, the SV ensemble was ultimately selected due to its enhanced robustness. Unlike a single model, the ensemble relies on the consensus of three high-performing yet diverse models. This consensus approach does not necessarily improve binary classification accuracy, but it provides a more reliable estimation of prediction probabilities. In particular, since the ensemble probability is computed from the weighted outputs of the three constituent models, disagreement among them results in a lower final probability. This feature becomes especially valuable when ranking or prioritizing candidates based on their predicted probability of activity, offering a more nuanced and cautious interpretation of borderline cases. For these reasons, the SV ensemble was selected as the preferred model for the qualitative prediction of human DPP4 inhibition.

To assess the reliability of predictions within the explored chemical space, the applicability domain (AD) approach described in the Section 4 was employed. This method revealed that 974 out of 984 molecules (99%) of the VS. were within the AD, confirming that the model operates reliably across most compounds in this validation dataset.

### 2.2. Feature Selection, Regression Model Development, and Optimization for Predicting DPP4 Inhibition (IC_50_)

**Figure 3:**
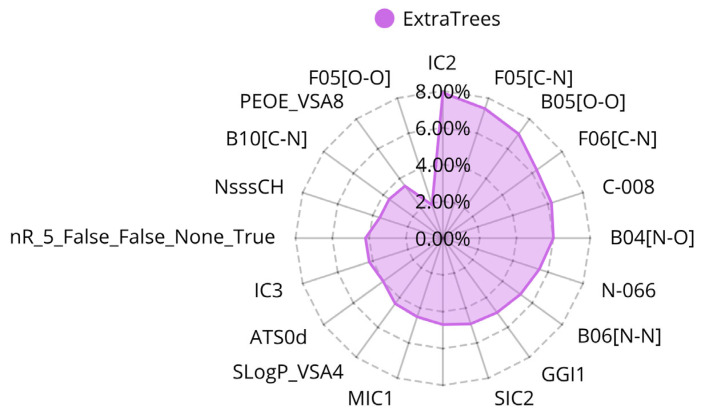
Radial plot of the feature importances of the descriptors for the QSAR regression model.

Following a similar approach to that used for classification, the regression models were developed using the GA for feature selection. The training set (TS) consisted of 2873 compounds, and an Extra Trees regression model utilizing 20 molecular descriptors was identified as the optimal solution (see Figure 3 and Table 4). After selection, the model’s hyperparameters were fine-tuned, and its predictive performance was evaluated through a 10-fold cross-validation using the VS of 960 compounds.

**Figure 4:**
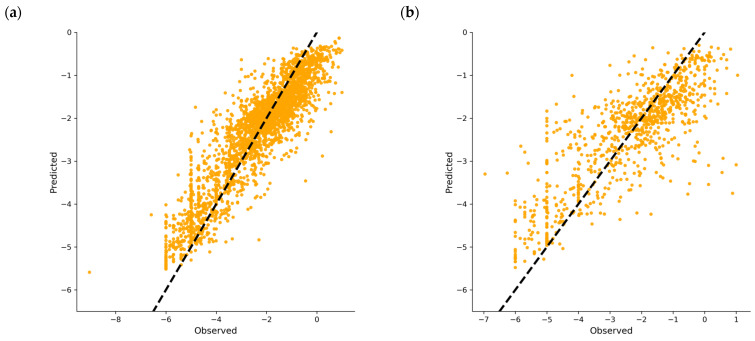
Observed vs. predicted plots of TS (**a**) and VS. (**b**). y = x line represented in black discontinued line, orange dots represent each molecule value.

The model exhibited optimal performance on the TS, achieving a mean R^2^ of 0.82 ± 0.002 across the different folds. For the VS, the model maintained robust predictive ability, with an average R^2^ of 0.67 ± 0.015, indicating acceptable generalizability to unseen data. Observed vs. predicted plots of the model are shown in Figure 4.

To assess the reliability of the model’s predictions across chemical space, AD was evaluated. A total of 911 compounds (95%) from the vs. were found to fall within the model’s AD, supporting the reliability of the predictions for the majority of cases. The remaining 49 compounds (5%) were classified as outside the AD and should, therefore, be interpreted with caution in terms of predictive confidence.

Overall, the regression model demonstrated strong predictive power and wide applicability within the chemical space of known DPP4is, reinforcing its value as a quantitative tool for estimating inhibitory potency.

### 2.3. DPPPRED-IV Web Server

**Figure 5:**
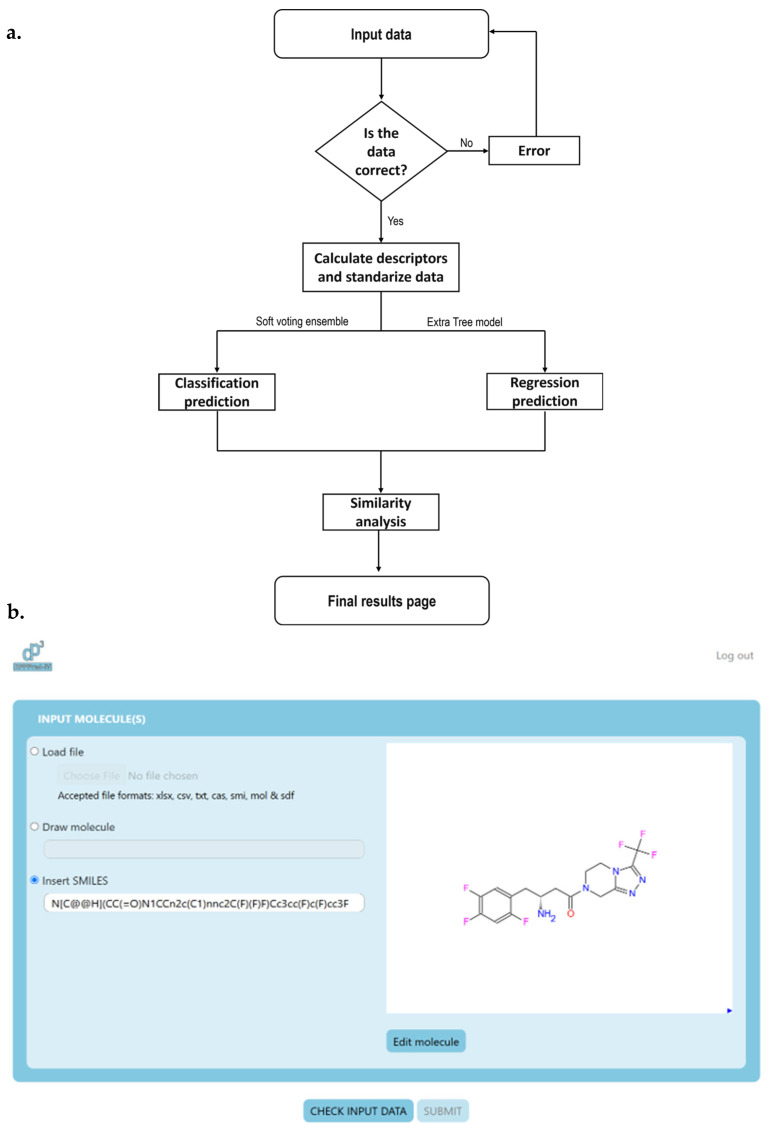
(**a**) Designed workflow of the DPPPRED-IV server; (**b**) Input screen of DPPPRED-IV web server showing sitagliptin molecules as input.

The operational workflow of the DPPPRED-IV web server (accessible at https://chemopredictionsuite.com/DPPPredIV, accessed on 8 June 2025) is illustrated in Figure 5a. Users can submit input data via two methods: by directly entering SMILES strings into a designated text field, directly drawing the molecule, or by uploading a file containing multiple SMILES codes. The platform supports several file formats, including .xlsx, .csv, and .txt (Figure 5b).

Once the input is provided, the server performs an initial structural validation to ensure the chemical integrity of the submitted molecules. Invalid entries trigger an automatic error message, prompting users to correct the input. If the structures are validated successfully, the platform proceeds to compute molecular descriptors, followed by data standardization, preparing the molecules for model-based evaluation.

The standardized data is then processed through the integrated classification models, which predict the likelihood of each molecule acting as a DPP4 inhibitor. After classification, the molecules are further evaluated using the regression model, which estimates their inhibitory activity in quantitative terms. The server also assesses predictive reliability, providing users with both classification and regression results along with a robust assessment of chemical space, enhancing the interpretability and utility of the results.

### 2.4. Case Study: Application of DPPPRED-IV to the MolPort Database

To assess the real-world applicability of the models implemented in the DPPPRED-IV server, we performed experimental validation using 29 structurally diverse compounds selected from the MolPort catalog (https://www.molport.com, accessed on 15 May 2022). These compounds were chosen following a virtual screening of the entire MolPort database using the DPPPRED-IV server and subsequently tested in vitro, as described in the Materials and Methods Section 4.11.

Each compound, along with the positive control (sitagliptin), was evaluated at a fixed concentration of 1.5 µM. Compounds were classified as experimentally active if they exhibited greater than 15% inhibition of DPP4 activity. The comparison between predicted and experimental results is summarized in Figure 3, providing insight into the server’s hit-identification capability during early-stage screening.

**Figure 6:**
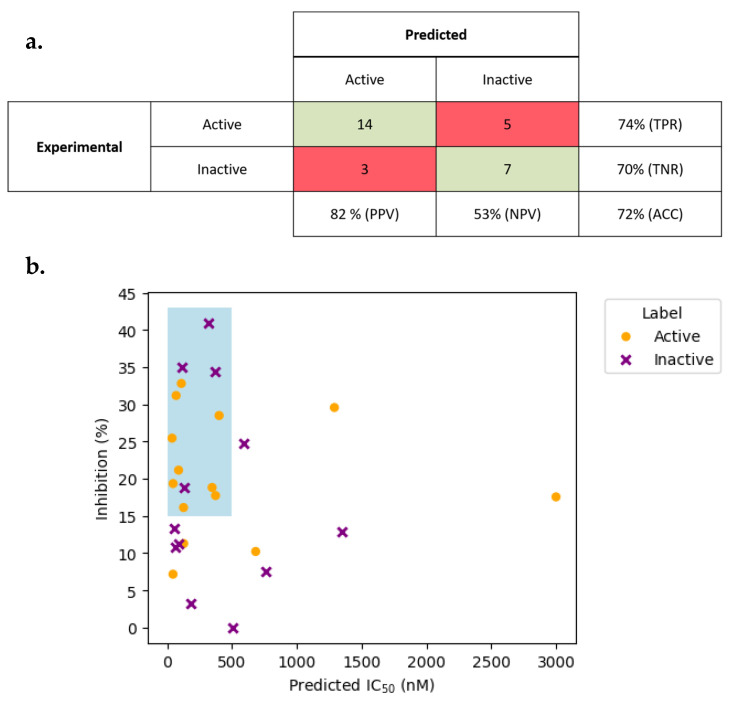
(**a**) Confusion matrix summarizing the classification performance of DPPPRED-IV on 29 MolPort compounds, using a 15% inhibition cutoff at 1.5 µM to define “active”. Cells are colored by outcome: true positives/negatives (green) and false positives/negatives (red). PPV: positive predictive value; NPV: negative predictive value; TPR: true positive rate; TNR: true negative rate; ACC: overall accuracy. (**b**) Scatter plot of QSAR-predicted IC_50_ (nM) versus experimentally measured inhibition (%) for the same compounds. Orange circles denote compounds predicted active (IC_50_ ≤ 50 nM), and purple crosses denote predicted inactives. The lightly shaded box (0–500 nM, 15–25% inhibition) highlights the early-stage hit-finding window in which the model enriches promising scaffolds.

Based solely on the classification output (Figure 6a), out of the 29 compounds tested, 19 were considered active (+), having inhibited the DPP4 activity by more than 15% (Supplemental Table S1). These hits represent promising candidates for further exploration of pharmaceutical developments. From a predictive standpoint, 17 of the 29 compounds had been identified as active by the DPPPRED-IV classification model. Among these, 14 were confirmed experimentally, yielding a hit identification rate (or PPV) of 82%. Focusing solely on the 19 experimentally active compounds, 14 were correctly predicted by the server, corresponding to a 74% success rate in true positive prediction. As a general metric, this validation yielded 72% of global accuracy.

If both predictive models are combined (Figure 6b) and considering only the compounds that fall within the AD for classification and regression models, each point maps a compound’s QSAR-predicted IC_50_ (*x*-axis) against its single-point inhibition at 1.5 µM (*y*-axis). The orange circles, compounds predicted as active (IC_50_ ≤ 50 nM), cluster predominantly at lower predicted potencies and generally yield higher inhibition values, with most lying above the 15% cutoff. By contrast, the purple crosse, predicted inactives, scatter toward higher IC_50_ predictions and often fall below the 15% line. Within the shaded box (0–500 nM, 15–25% inhibition), nine orange circles occupy this early-hit window compared to only five purple crosses, illustrating how DPPPRED-IV enriches for measurable inhibitors. A handful of outliers, such as a predicted inactive at ~315 nM showing ~41% inhibition and a predicted active at ~3000 nM with ~18% inhibition, highlight the inherent noise of single-point assays but do not impair the overall trend.

These results validate the robustness and practical relevance of the QSAR models implemented in DPPPRED-IV, demonstrating their value as an effective pre-screening tool for identifying potential DPP4is from large compound libraries.

## 3. Discussion

As the global prevalence of T2DM continues to rise, the need for effective therapeutic strategies to maintain glycemic control and prevent associated complications is more urgent than ever. Although established treatments such as metformin, GLP-1 receptor agonists, insulin injections, and DPP4is are widely used in clinical practice, patient response varies significantly. This variability is likely due to the multifactorial and heterogeneous nature of T2DM, including complex interactions with the gut microbiota [52,53,54,55,56,57].

With this purpose, in this work, we have developed a suite of QSAR models aimed at predicting the potential inhibitory activity of small molecules against human DPP4. The study included four models in total: three classification models and one regression model. Descriptor selection for each model was performed using a GA, which facilitated the identification of reduced but informative subsets of molecular features capable of yielding accurate and interpretable models.

The analysis of selected descriptors revealed insightful structural features linked to DPP4 inhibition. In the classification models, descriptor contributions were relatively balanced (ranging from 12% to 14%). A key descriptor present across all three models was SlogP_VSA2, which captures the contribution of hydrophobic surface area to the molecule’s logP value (octanol/water partition coefficient). This descriptor highlights the importance of hydrophobicity in promoting interactions with the DPP4 binding pocket. This particular relevance of hydrophobicity for the DPP4 binding pocket aligns well with previous structural works that suggested the relevance of the hydrophobic regions of the pocket to interact with known inhibitors [58]. Another shared descriptor, IC2, corresponds to a second-order neighborhood symmetry index, indicating that molecular topology contributes meaningfully to activity, representing a property that influences ligand fit and potential interaction within protein pockets. Additionally, descriptors F04[C–N] and F05[C–N], found in two of the three models, represent the presence of C–N fragments at specific topological distances, potentially capturing hydrogen bond donors or acceptors that contribute to DPP4-ligand binding through polar or electrostatic interactions [59].

In the regression model built with the ExtraTrees algorithm, the most influential features included IC2 (7.9%), F05[C–N] (7.42%), B05[O–O] (7.01%), and F06[C–N] (6.27%). F05[C–N] and F06[C–N] are consistent with the previously noted importance of nitrogen atoms flanked by carbon atoms, which could be related to the presence of B05[O–O], stating the relevance of oxygen and nitrogen atoms which are crucial for interactions due to their ability to form hydrogen bonds within the binding pocket, which enhances binding affinity and specificity. In concordance with classification models, IC2 presented relevance, which supports the idea that the molecular topology contributes to the activity.

Future investigations could evaluate the incorporation of other structural descriptors and quantum-chemical parameters to assess whether these additional features can further improve the predictive performance and expand the chemical space coverage of our QSAR models.

Numerous QSAR models for predicting DPP4 inhibition have been published in recent years, as summarized in Table 5. When comparing our classification models to those reported in the literature, such as the study by Cai et al. [47], which achieved an accuracy of 87% using 1743 compounds, our ensemble-based classification model yielded slightly lower accuracy (80%). However, unlike Cai et al., who excluded all compounds with IC_50_ values between 50 nM and 500 nM, our model leveraged the full chemical diversity available in the ChEMBL dataset. This decision resulted in broader chemical coverage and significantly improved generalizability, with 99% of validation compounds falling within the applicability domain, which is supposed to be an important advantage, especially considering that comparable studies often do not explicitly assess or report applicability domain coverage.

In terms of regression performance, several previous studies have reported high R^2^ values, such as Gong et al. [48] (R^2^ = 0.90 with 61 compounds), Al-Fakih et al. [49] (R^2^ = 0.94 with 134 compounds), and Buiu et al. [51] (R^2^ = 0.85 with 35 compounds). However, these models were trained on relatively small datasets, which may limit their applicability to structurally diverse compounds. In contrast, our regression model was developed from a curated dataset of 3834 compounds, providing both solid predictive power (R^2^ = 0.67) and a broad applicability domain that covers 95% of the validation compounds. On the other hand, Ma et al. [50] used a significantly larger dataset (>8000 compounds), but the resulting model achieved a much lower predictive performance (R^2^ = 0.23), likely due to dataset heterogenicity, including data from multiple species and varying assay conditions that introduced substantial noise.

To ensure the practical application of the models developed, we implemented them in a web-based platform called DPPPRED-IV, integrated within the ChemoPredictionSuite (https://chemopredictionsuite.com/). This platform provides users with an accessible and intuitive interface for uploading molecular structures and receiving predicted DPP4 inhibition values. Importantly, the server supports non-expert users, facilitating the integration of QSAR models into real-world compound screening workflows.

To study the predictive performance of the platform, we performed a virtual screening of 8384 compounds from the MolPort catalog using the QSAR predictive models developed for DPP4 inhibition. From this screen, 29 candidate compounds were prioritized based on diverse predicted DPP4 inhibitory activities rather than the highest-scoring hits to ensure a diverse set. These compounds were subsequently evaluated in vitro, where 19 demonstrated measurable DPP4 inhibition above 15% at a concentration of 1.5 µM; out of these 19 active compounds, 14 were correctly predicted by DPPRED-IV. In the enzymatic validation, a 15% inhibition cutoff at 1.5 µM was applied as a primary triage filter to detect moderate-affinity scaffolds. Application of a more stringent 50% threshold would have yielded no qualifying hits at this concentration despite its closer alignment with classical IC_50_ definitions. Such lower single-point cutoffs (10–30% inhibition) are standard in early-stage screening to balance assay variability and predictive uncertainty, thereby preserving chemical diversity for downstream dose–response studies and lead optimization [60,61,62,63].

This strategy aligns with precedents in DPP4 inhibitor discovery: Li et al. reported inhibitors with IC_50_ values of 5–50 µM using low-micromolar activity benchmarks [64], and Montes demonstrated that scaffolds exhibiting 25% inhibition at 500 µM can serve as viable starting points for optimization [65]. Modest initial activity has, therefore, been shown to indicate follow-up potential. However, future studies should incorporate systematic dose–response profiling and assessment of alternative inhibition thresholds to refine the sensitivity–specificity balance.

In addition to binary classification at the 50 nM activity threshold, DPPPRED-IV offers continuous IC_50_ predictions to help rank compounds by expected potency. In Figure 6b, we highlight the 0–500 nM/ > 15 inhibition window, commonly used to capture moderate-affinity hits and observe nine compounds within this region versus only five outside it. This enrichment suggests that using both the classification label and the regression value steers users toward a higher proportion of true actives than random selection, thereby streamlining early-stage prioritization in DPP4 inhibitor discovery.

For example, compounds 24 and 25 (Table S1) were correctly flagged as active and have regression-predicted IC_50_ values of 38.5 nM and 46.2 nM, respectively, underscoring their lead potential. A handful of outliers, such as a predicted inactive at 315 nM achieving 40% inhibition, reflect single-point assay variability but do not obscure the overall trend: lower predicted IC_50_ values generally correspond to higher observed inhibition. Together, these results confirm that the regression output adds a valuable, potency-driven dimension to the binary QSAR classification, enhancing decision making in hit selection.

Moreover, recent studies have underscored the potential role of microbial DPP4 homologs in modulating host glucose homeostasis and influencing T2DM progression [65,66,67,68]. Although the present work did not directly assess host–microbiota DPP4 interactions, it is worth noting that next-generation DPP4 inhibitors might benefit from incorporating microbiome-related insights. As a future perspective, DPPPRED-IV predictions could be coupled with structure-based docking against representative bacterial DPP4 enzymes, following protocols similar to those in previous work [66], to prioritize compounds likely to retain efficacy in the context of gut microbiota. Such an integrated pipeline would more accurately reflect the complexity of microbiota-mediated effects on T2DM pharmacotherapy and help guide the design of more effective microbiome-aware inhibitors.

This indicates that the DPPPRED-IV server provides meaningful prioritization of candidates and reinforces the platform’s value as an effective computational tool for large-scale screening in early-phase drug discovery. However, it is important to acknowledge that the current validation set is relatively limited in size, which may constrain the experimental evaluation of the model’s broader applicability. Expanding the dataset with new experimental results will be essential for improving the robustness of the models and refining the predictive capabilities of the DPPPRED-IV server. In addition, we used single-concentration in the enzymatic assays to experimentally confirm the DPP-4 inhibitory activity. The future characterization should include comprehensive dose–response analyses (e.g., IC_50_ determination). As more data become available, the system will be better positioned to guide early-stage discovery efforts with increased confidence.

## 4. Materials and Methods

### 4.1. Data Collection and Curation

A dataset of DPP4is was collected from the ChEMBL database (https://www.ebi.ac.uk/chembl/, accessed on 12 January 2025) [66] and curated according to the following criteria: (i) only assay data targeting human DPP4 were included (ChEMBL code: 284); and (ii) only experimental IC_50_ values were retained. This initial curation resulted in a dataset of 5386 compounds, with IC_50_ values ranging from 0.012 nM to 1.1 M.

For qualitative model building, an IC_50_ threshold of ≤ 50 nM was used to label compounds as “active,” whereas those with IC_50_ > 50 nM were deemed “inactive,” in line with the benchmark set by Cai et al. [47]. This stringent cutoff reflects widely accepted lead-likeness criteria in medicinal chemistry, where sub-50 nM potency is taken as the optimal threshold for high-affinity interactions and efficient downstream progression. In contrast to Cai et al. [47] work and to preserve dataset comprehensiveness, compounds with intermediate activities (50–500 nM) were still retained.

For quantitative model development, the whole retrieved dataset with the IC_50_ values was filtered, selecting those that presented an exact value of IC_50_ (marked with “=” in the database), yielding a total of 5225 compounds with IC_50_ values ranging from 0.012 nM to 1.1 M.

To ensure the quality and reliability of the datasets used for QSAR model development, a rigorous data curation protocol was followed. This included:

a. Removal of salts and waters: All associated salts and water molecules were removed to ensure chemical consistency and eliminate confounding molecular components.
b. Duplicate handling: A comprehensive check for duplicates was performed to prevent redundancy and bias:
  i. For duplicates with identical response values (binary classification or IC_50_) values, only one entry was retained.

- In the case of the regression dataset, for duplicates with slightly different IC_50_ values (within a predefined threshold of standard deviation/mean < 0.2), the geometric mean was calculated and used to represent the compound’s activity. Moreover, a log transformation is performed in order to obtain pIC_50_.
  i. If the variation between IC_50_ values exceeded the threshold, the corresponding entries were excluded from the dataset to preserve data integrity.

These steps were essential to maintain the robustness of the modeling process and to ensure that both qualitative and quantitative models were built upon accurate and harmonized datasets, yielding a curated dataset of 3929 and 3834 compounds, respectively.

### 4.2. Feature Calculation

A total of 4676 molecular descriptors were calculated for each compound using WOTAN (v 1.0), an in-house software developed by ProtoQSAR (Valencia, Spain). This tool is implemented in Python (v. 3.9.4.) [67] and integrates functionalities from widely used chemoinformatics libraries, including RDKit (v. 2021.03.2.) [68] and Mordred (v. 1.2.0.) [69], along with additional custom descriptor sets based on literature [70]. The calculated descriptors encompass a wide range of physicochemical, topological, geometrical, constitutional, and electronic features and are organized into 20 descriptor categories, as detailed in Table 6.

### 4.3. Initial Feature Reduction

For the development of all QSAR models, an initial unsupervised feature selection and preprocessing pipeline was applied to the complete dataset to ensure data quality and enhance model performance. This process involved several sequential steps:

1. Removal of constant and infinite values: Descriptors with constant values across all compounds or containing infinite values were removed, as they do not contribute to compound differentiation and may compromise model robustness.
2. Elimination of highly correlated descriptors: To reduce redundancy and mitigate multicollinearity, pairwise Pearson correlation coefficients were computed across all descriptors. When two descriptors showed a correlation greater than 0.90, only one was retained.

Following descriptor filtering, missing values in the descriptor matrix were imputed using the k-Nearest Neighbors (kNN) method, implemented with k = 3 and uniform weights [71]. This approach preserves the internal structure of the dataset while effectively managing missing data.

Finally, all datasets were normalized using the Standard Scaler technique [72], which centers the data by removing the mean and scales it to unit variance. This step ensures that all descriptors contribute equally to model training, regardless of their original scale or magnitude.

### 4.4. Train and Validation Set Splits

The datasets used for QSAR model development were divided into a TS comprising 75% of the compounds and a VS comprising the remaining 25%. To ensure a balanced and representative distribution of chemical space across both sets, a semi-random stratified splitting approach was applied using a k-means clustering algorithm [73].

This method involved clustering the dataset based on molecular descriptors prior to splitting, thereby ensuring that compounds from each cluster were proportionally assigned to both the TS and VS. This strategy preserved the chemical diversity of the dataset and contributed to a more robust and generalizable model by reducing the risk of overfitting to specific structural patterns.

### 4.5. Feature Selection and Model Generation

Feature selection and model generation were carried out using a GA-based approach designed to identify optimal subsets of molecular descriptors for QSAR modeling. The GA began by randomly selecting 50% of the available descriptors to form the initial population of descriptor subsets. Each subset was used to build a QSAR model using the TS, evaluated through five-fold internal cross-validation.

Models were ranked based on their performance scores: F1-score for classification models and R^2^ for regression models. To encourage model simplicity and reduce overfitting, a penalty was applied based on the number of descriptors used. This scoring scheme ensured that models with fewer descriptors were favored, provided they maintained high predictive performance.

**Figure 7:**
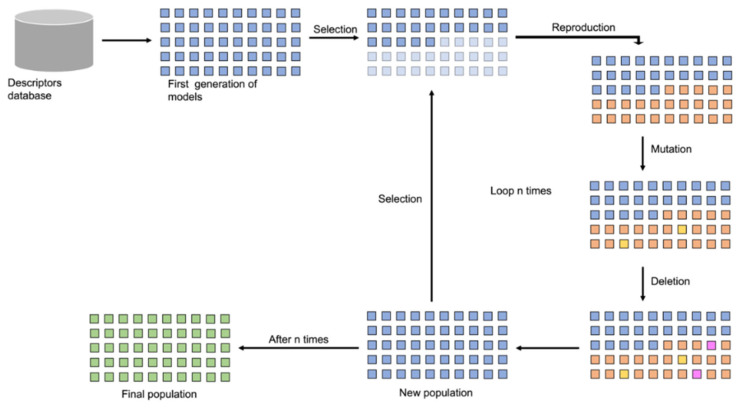
Schematic representation of the GA employed. Squares are colored by subset stage: blue for initial, orange for child, yellow for mutated, pink for deleted, and green for final descriptor subsets.

After ranking, the top 50% of descriptor subsets were selected as “parents” to generate a new population. For each pair of parents, two new “child” subsets were created by recombining half of the descriptors from each parent. Genetic variation was introduced via mutation (30% probability), replacing one descriptor with a randomly selected new one, and deletion (30% probability), removing one descriptor from the subset. This evolutionary process was repeated for 200 generations (default stopping criterion), resulting in an optimized population of descriptor subsets. A schematic representation of this process is provided in Figure 7.

Model development was performed in Python [67], using the scikit-learn framework [74]. For both classification and regression tasks, different ML algorithms were applied, selecting the ones that resulted in models with better performance.

### 4.6. Hyperparameter Tunning

To maximize the performance of the ML models developed in this study, hyperparameter optimization was carried out using the GridSearchCV function [75] from the scikit-learn library (v. 1.0.2.) [74]. This method conducts an exhaustive search over a predefined grid of hyperparameter values, systematically evaluating every possible combination.

For each configuration, model performance was assessed using ten-fold cross-validation, ensuring that the evaluation was both robust and generalizable. This approach allowed for the identification of the optimal hyperparameter set that achieved the best average cross-validation score for each algorithm.

The ranges of hyperparameter values were strategically defined based on prior domain knowledge and preliminary empirical testing. Hyperparameter sets that consistently yielded the highest performance across iterations were selected as the final configurations for each model. This tuning process was applied to all classification and regression models included in the study.

### 4.7. QSAR Performance Evaluation

To assess the predictive performance and generalizability of the selected models, the performance metrics defined in Equations (1)–(9) were computed using true positive (*TP*), true negative (*TN*), false positive (*FP*), and false negative (*FN*), for classification purposes and observed value ($y_{i})$, predicted value (${\overset{ˇ}{y}}_{i}$) for regression. The mean values and standard deviations across all ten folds were reported. This approach allowed for a comprehensive evaluation of model stability and reliability.

(1) $$Accuracy=\frac{TP+TN}{TP+TN+FN+FP}$$

(2) $$Recall=\frac{TP}{TP+FN}$$

(3) $$Precision=\frac{TP}{TP+FP}$$

(4) $$Specificity=\frac{TN}{TN+FP}$$

(5) $$FPR=\frac{FP}{TN+FP}$$

(6) $$FNR=\frac{FN}{TP+FN}$$

(7) $$MCC=\frac{TP\times TN-FP\times FN}{\sqrt{\left(TP+FN)(TP+FP)(TN+FN)(RN+FP\right)}}$$

(8) $$F1\;score=2\times \frac{Precision\times Recall}{Precision+Recall}$$

(9) $$R^{2}=1-\frac{\sum_{i}{\left(y_{i}-{\overset{ˇ}{y}}_{i}\right)}^{2}}{\sum_{i}{\left(y_{i}-{\bar{y}}_{i}\right)}^{2}}$$

### 4.8. Ensemble Expert System

For the classification models developed to predict DPP4 inhibitory activity, an ensemble modeling strategy was employed to enhance predictive performance and robustness with different QSAR models.

Both HV (majority rule) and SV (averaging predicted probabilities) ensemble techniques were implemented to combine the outputs of selected base classifiers. In the case of SV, model-specific weights were optimized to maximize prediction reliability, assigning greater influence to models that improve the performance of the ensemble prediction. This aggregation of model decisions aimed to reduce individual model bias and variance, ultimately improving the robustness of the final classification predictions for DPP4 inhibition

### 4.9. Applicability Domain

The AD of the developed QSAR models was determined following the methodology proposed by Sahigara et al., which is based on a kNN approach combined with an adaptive kernel estimation of the probability density function [76]. This method enables the identification of regions in the descriptor space where the model predictions are considered reliable.

The process begins by defining a set of thresholds for each training compound derived from the distribution of distances to its nearest neighbors. These thresholds are then used to establish a decision rule that determines whether a new compound falls within the model’s AD.

In the case of the ensemble SV models, this AD assessment was applied to each individual base model within the ensemble. For every query compound, a reliability score was calculated per model, reflecting the compound’s similarity to the training data; if the compound was within the AD for an individual model, a score of 0.33 was retrieved for each of the models. A compound was considered to fall within the ensemble’s AD if the sum of individual model scores exceeded 0.6, which means falling within the AD for two or more of the models, indicating a sufficient level of confidence in the aggregated prediction.

### 4.10. Web Server Development

The developed QSAR models were implemented in a dedicated module DPPRED-IV within a web-based computational platform called ChemoPredictionSuite (https://chemopredictionsuite.com/), built using the Django framework (v. 2.1.5.) [77] in Python [67]. This platform offers a user-friendly interface that enables users to submit molecular structures, typically via SMILES input, and receive predicted values for the modeled properties in real-time.

The aim of the integration of the predictive models into this web server is to enhance their accessibility, usability, and scalability, making them readily available to researchers and practitioners without the need for local installation or advanced programming skills. This deployment supports the widespread application of the models in drug discovery and related areas, facilitating the translation of QSAR-based predictions into practical workflows.

### 4.11. DPP4 Enzymatic Activity Assay

The DPP4 inhibitory activity of 29 selected compounds was experimentally evaluated using one commercial DPP4 inhibitor screening assay Kit (ab133081, Abcam, Cambridge, UK), following the manufacturer’s instructions. These compounds were selected from a total of 8384 unique MolPort compounds from the “Biologically active compounds library” after a virtual screening with the QSAR models developed to predict DPP4 inhibitory capacity. The selection was made in order to have a diverse set whose predicted activities span a range of model outputs.

DPP4 enzymatic activity was assessed by monitoring the cleavage of a fluorogenic substrate, which generates a fluorescent signal (excitation = 360 nm, emission = 460 nm) proportional to enzymatic activity. Each compound was tested at a final concentration of 1.5 µM, and fluorescence was recorded kinetically over 30 min at 37 °C. Enzymatic activity was quantified by calculating the slope of fluorescence increase between minutes 10 and 20 using the equation:

(10) $$Slope=\frac{{FLU}_{min20}-{FLU}_{min10}}{T_{20}-T_{10}}=\frac{∆FLU}{minute}$$

The inhibitory activity of each compound was expressed as the percentage of relative inhibition, calculated using the equation:

(11) $$\%\;Inhibition=\frac{{Slope}_{control}-{Slope}_{sample\;inhibitor}}{{Slope}_{control}}\times 100$$

## 5. Conclusions

In this work, we developed and validated QSAR models to predict the inhibitory activity of small molecules against human DPP4. By combining qualitative and quantitative approaches and training on a diverse dataset from ChEMBL, our models cover a broad chemical space and show strong predictive performance.

These models were implemented in DPPPRED-IV, a user-friendly web server integrated into the ChemoPredictionSuite platform. Experimental validation confirmed the tool’s ability to identify potential DPP4is, demonstrating its value for early-stage screening in drug discovery. Overall, DPPPRED-IV provides an accessible and reliable resource to support the identification of new DPP4is of interest to the treatment of T2DM.

## Figures and Tables

**Table 1:** Characteristics of commonly used DPP4is.

DPP4i Name	Structure	Half-Life (h)	Absolute Bioavailability (%)	Dose	Plasma Protein Binding (%)	Metabolism	Elimination Route
Sitagliptin	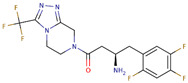	12.5	87	100 mg once daily	38	Minimal	Predominantly renal
Saxagliptin	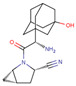	2.5	75	5 mg once daily	Negligible	Hydrolysis	Metabolism
Vildagliptin	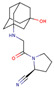	2	85	50 mg twice daily	9	Hydrolysis	Metabolism
Linagliptin	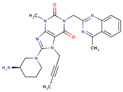	12	30	5 mg once daily	90	Minimal	Predominantly biliary
Alogliptin	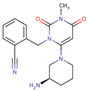	20	100	25 mg once daily	30	Minimal	Predominantly renal
Teneligliptin	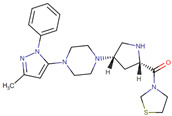	24.2	74	20 mg once daily	78	Minimal	Predominantly renal

**Table 2:** Descriptor information of the classification models with the % of importance.

Model	Descriptor	Feature Importance (%)	Description
Random Forest	GATS6s	11%	Geary autocorrelation of lag 6 weighted by I-state.
	MIC1	12%	1-ordered modified information content.
	R4u	12%	R autocorrelation of lag 4/unweighted.
	IC2	15%	Information Content index (neighborhood symmetry of 2-order).
	SLogP_VSA2	12%	MOE logP VSA Descriptor 2 (−0.40 ≤ x < −0.20).
	EEig02u	13%	Eigenvalue of order 2 from the edge adjacency matrix unweighted.
	TI2	12%	Second Mohar index.
Extra Trees	RBF	12%	Rotatable bond fraction
	VSA_EState2	9%	VSA EState Descriptor 2 (4.78 ≤ x < 5.00)
	HATS5s	10%	leverage-weighted autocorrelation of lag 5/weighted by I-state
	IC2	17%	Information Content index (neighborhood symmetry of 2-order)
	SLogP_VSA2	13%	MOE logP VSA Descriptor 2 (−0.40 ≤ x < −0.20)
	EEig02u	12%	Eigenvalue of order 2 from the edge adjacency matrix unweighted
	F04[C-N]	13%	Frequency of C-N at topological distance 4
	PEOE_VSA8	13%	MOE Charge VSA Descriptor 8 (0.00 ≤ x < 0.05)
AdaBoost	F05[C-N]	7%	Frequency of C-N at topological distance 5
	R4m	11%	R autocorrelation of lag 4/weighted by mass
	AATSC7v	13%	Averaged and centered Moreau-Broto autocorrelation of lag 7 weighted by vdw volume
	SLogP_VSA2	13%	MOE logP VSA Descriptor 2 (−0.40 ≤ x < −0.20)
	HATS8s	3%	Leverage-weighted autocorrelation of lag 8/weighted by I-state
	ATSC0c	23%	Centered Moreau-Broto autocorrelation of lag 0 weighted by gasteiger charge
	TI2	17%	Second Mohar index
	SLogP_VSA4	13%	MOE logP VSA Descriptor 4 (0.00 ≤ x < 0.10)

**Table 3:** Model performance for each model, individually and for the soft and hard voting ensemble systems.

	ML Algorithm	Accuracy (%)	Recall (%)	Precision (%)	Specificity (%)	FPR (%)	FNR (%)	F1-Score	MCC
TS (2945 compounds)	AdaBoost	81 ± 0.3	71 ± 0.9	78 ± 0.1	87 ± 0.1	13 ± 0.1	29 ± 0.9	0.74 ± 0.006	0.59 ± 0.007
	Extra Trees	94 ± 0.4	90 ± 0.6	93 ± 0.5	96 ± 0.3	4 ± 0.3	10 ± 0.6	0.92 ± 0.005	0.87 ± 0.009
	Random Forest	87 ± 0.4	82 ± 0.5	84 ± 0.9	91 ± 0.6	9 ± 0.6	18 ± 0.5	0.83 ± 0.005	0.73 ± 0.009
	HV	89 ± 0.3	84 ± 0.4	87 ± 0.4	92 ± 0.3	8 ± 0.3	16 ± 096	0.85 ± 0.004	0.76 ± 0.006
	SV	92 ± 0.2	88 ± 0.6	90 ± 0.3	94 ± 0.2	6 ± 0.2	12 ± 0.6	0.89 ± 0.003	0.83 ± 0.005
VS (984 compounds)	AdaBoost	73 ± 1	60 ± 2.4	66 ± 1.2	81 ± 0.8	19 ± 0.8	40 ± 2.4	0.63 ± 0.02	0.42 ± 0.022
	Extra Trees	80 ± 0.6	69 ± 1.6	77 ± 1.1	87 ± 0.9	13 ± 0.9	31 ± 1.6	0.73 ± 0.009	0.57 ± 0.012
	Random Forest	77 ± 1	69 ± 3.1	71 ± 0.6	82 ± 0.8	18 ± 0.8	31 ± 3.1	0.70 ± 0.02	0.51 ± 0.021
	HV	79 ± 2	68 ± 3	75 ± 3	86 ± 2	14 ± 2	32 ± 3	0.71 ± 0.02	0.55 ± 0.037
	SV	80 ± 1	69 ± 3	76 ± 3	87 ± 2	13 ± 2	31 ± 3	0.73 ± 0.01	0.57 ± 0.02

FPR: False Positive Rate; FNR: False Negative Rate; MCC: Matthews Correlation Coefficient; TS: Training Set; VS: Validation Set; HV: Hard Voting; SV: Soft Voting.

**Table 4:** Descriptor information of the regression models with the % of importance.

Model	Descriptor	Feature Importance (%)	Description
Extra Trees	IC2	7.9%	Information Content index (neighborhood symmetry of 2-order)
	F05[C-N]	7.4%	Frequency of C–N atom pairs at topological distance 5
	B05[O-O]	7.0%	Presence/absence of O–O atom pairs at topological distance 5 (burden matrix)
	F06[C-N]	6.3%	Frequency of C–N atom pairs at topological distance 6
	C-008	6.2%	Atom-centered fragment: sp2 carbon connected to electronegative atoms
	B04[N-O]	6.0%	Presence/absence of N–O atom pairs at topological distance 4
	N-066	5.5%	Atom type E-state index for tertiary amine nitrogen
	B06[N-N]	5.2%	Presence/absence of N–N atom pairs at topological distance 6
	GGI1	5.0%	Topological charge index of order 1
	SIC2	4.9%	Structural information content index of order 2
	nR_5_False_False_False_True	4.7%	Number of 5-membered rings matching specific structural criteria
	MIC1	4.5%	1-ordered modified information content
	SLogP_VSA4	4.4%	MOE logP VSA Descriptor 4 (interval defined by logP contribution)
	ATS0d	4.0%	Autocorrelation of lag 0 weighted by sigma electrons
	IC3	4.2%	Information Content index (neighborhood symmetry of 3-order)
	nR_5_False_False_None_True	4.2%	Number of 5-membered rings with specific heteroatom and aromaticity pattern
	NsssCH	3.6%	Atom type E-state: carbon with three single bonds to saturated atoms
	B10[C-N]	3.6%	Presence/absence of C–N atom pairs at topological distance 10 (burden matrix)
	PEOE_VSA8	3.5%	MOE Charge VSA Descriptor 8 (0.00 ≤ x < 0.05)
	F05[O-O]	1.9%	Frequency of O–O atom pairs at topological distance 5

**Table 5:** Comparison of the different predictive models for inhibition against human DPPIV available in the literature in the last ten years.

Type	Model	Performance for the Test Set	Dataset Size
Classification	DPPPRED-IV	Accuracy: 80%	3929 compounds
	Cai et al. [47]	Accuracy: 87%	1743 compounds
Regression	DPPPRED-IV	R^2^: 0.67	3834 compounds
	Gong et al. [48]	R^2^: 0.90	61 compounds
	Al-Fakih et al. [49]	R^2^: 0.94	134 compounds
	Ma et al. [50]	R^2^: 0.23	8327 compounds
	Buiu et al. [51]	R^2^: 0.85	35 compounds

**Table 6:** Descriptor groups present in the WOTAN script.

Descriptors Groups in WOTAN	
Autocorrelations	Functional Groups
Bidimensional	Connectivity indexes
Topological charge	Information indexes
Atom centered	Molecular properties
Constitutionals	Rdkit 3D
CPSA (Charged Partial Surface Area)	Type MOE
Edge Adjacency	Topological
Electro topological estate	Burden Eigenvalues
Physicochemical	Eigenvalues
Getaway	Walk Path Counts

## Data Availability

The original contributions presented in this study are included in the article/Supplementary Material. Further inquiries can be directed to the corresponding author.

## Supplementary Materials

The following supporting information can be downloaded at: https://www.mdpi.com/article/10.3390/ijms26125579/s1.

## Abbreviations

The following abbreviations are used in this manuscript:

AD	Applicability Domain
GA	Genetic algorithm
HV	Hard voting
DPP4	Dipeptidyl peptidase-4
DPP4is	DPP4 inhibitors
FNR	False Negative Rate
FPR	False Positive Rate
GIP	Glucose-dependent insulinotropic polypeptide
GLP-1	Glucagon-like peptide-1
kNN	k-Nearest Neighbors
MCC	Matthews Correlation Coefficient
ML	Machine learning
PPV	Positive Predicted Value
PYY	Peptide Tyrosine-Tyrosine
QSAR	Quantitative Structure–Activity Relationship
SGLT2	Sodium-glucose transport protein 2
SV	Soft voting
T1DM	Type 1 diabetes mellitus
T2DM	Type 2 diabetes mellitus
TS	Training set
VS	Validation set
