# Supplementary material for: DPPPRED-IV: An Ensembled QSAR-Based Web Server for the Prediction of Dipeptidyl Peptidase 4 Inhibitors
Source: Int J Mol Sci. 2025 Jun 11;26(12):5579. doi: 10.3390/ijms26125579 (PMC12192733; doi:10.3390/ijms26125579)
Supplement: Supplementary file 1 [file ijms-26-05579-s001.zip › Supplemental_DPPPRED-IV.pdf]

Table S1. Experimental validation results (green indicates positive (+) and red indicates negative (-)).

ID	SMILES	Representation	Exp. binary	Pred. binary	Pred. IC_{50} (nM)
1	Cc1ccc(cc1)S(=O)(=O)c1nnn2c1nc(N1CCN(CC1)c1ccccn1)c1ccccc21	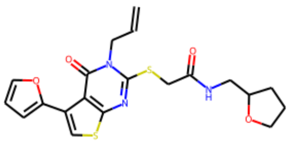	+	+	148.1
2	CCc1ccc(cc1)S(=O)(=O)c1nnn2c1nc(N1CCCC(C)C1)c1cc(Cl)ccc21	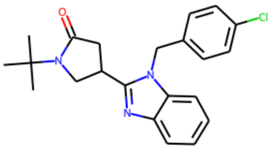	+	-	372.3
3	COCCn1c2nc3ccccn3c(=O)c2cc(c1=N)S(=O)(=O)c1ccc(F)cc1	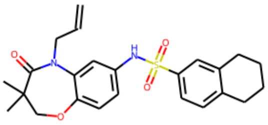	-	-	1347.9
4	COCCn1c2nc3c(C)cccn3c(=O)c2cc(c1=N)S(=O)(=O)c1ccc(F)cc1	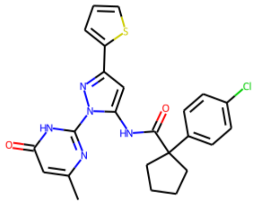	-	-	52.2
5	CCOc1ccc(cc1)-n1c(SCC(=O)Nc2cc(C)on2)nnc1-c1c[nH]c2ccccc12	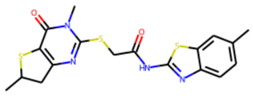	+	+	401.2

6	COc1ccccc1-c1nnc(NC(=O)c2ccc(cc2)S(=O)(=O)N(C)CC2CCCO2)o1	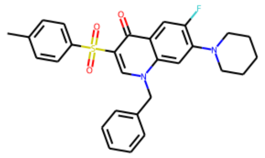	-	-	505.9
7	Cc1cc(=O)[nH]c(n1)-n1nc(cc1NC(=O)C1(CCCC1)c1ccc(Cl)cc1)-c1cccs1	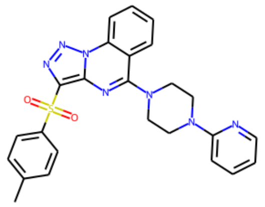	+	+	347.5
8	COc1ccc(CN2CCN(CC2)c2ncnc3sc4CCC(C)Cc4c23)cc1F	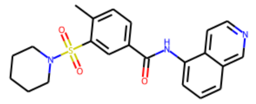	+	+	3000.2
9	COc1cccc(c1)-c1nc2Oc3c(C)ncc(CO)c3Cc2c(SCc2ccccc2)n1	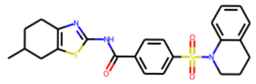	+	-	118.4
10	COC(=O)CSc1nnc(CNC(=O)c2ccc(cc2)S(=O)(=O)N2CCCCC2)n1C	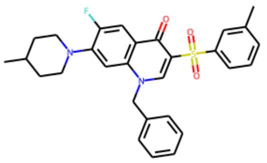	+	-	594.3
11	C=CCn1c(SCC(=O)NCC2CCCO2)nc2scc(-c3ccco3)c2c1=O	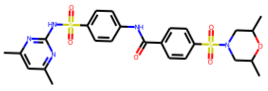	-	-	86.7

12	COc1ccc(cc1)-c1csc2nc(SCC(=O)NCC3CCCO3)n(CC=C)c(=O)c12	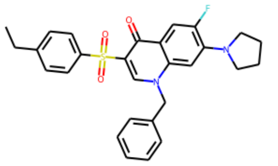	+	-	315.2
13	CC1CCc2nc(NC(=O)c3ccc(cc3)S(=O)(=O)N3CCCc4ccccc34)sc2C1	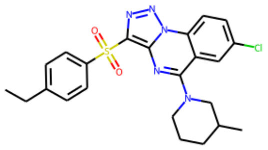	-	+	45.5
14	O=C(NCCc1c[nH]c2ccccc12)C1CCC(Cn2c(=S)[nH]c3ccccc3c2=O)CC1	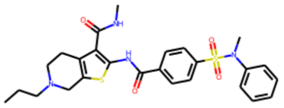	+	+	373.8
15	CC(C)(C)N1CC(CC1=O)c1nc2ccccc2n1Cc1ccc(Cl)cc1	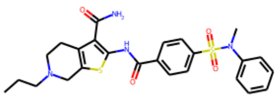	-	-	757.4
16	CC1Cc2nc(SCC(=O)Nc3nc4ccc(C)cc4s3)n(C)c(=O)c2S1	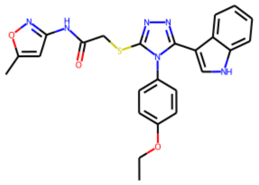	+	+	38.5
17	CN(C)CCCn1c2CCCc2c(SCC(=O)Nc2nc3ccc(C)cc3s2)nc1=O	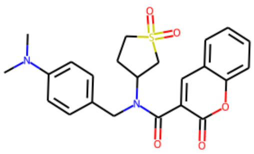	+	+	1290.4

18	CC1CN(CC(C)O1)S(=O)(=O)c1ccc(cc1)C(=O)Nc1ccc(cc1)S(=O)(=O)Nc1nc(C)cc(C)n1	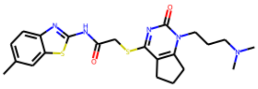	+	+	88.6
19	CCCN1CCc2c(C1)sc(NC(=O)c1ccc(cc1)S(=O)(=O)N(C)c1ccccc1)c2C(N)=O	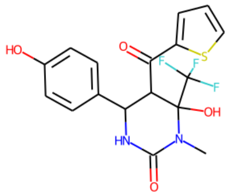	-	-	181.1
20	Cc1ccc(cc1)S(=O)(=O)c1cn(Cc2ccccc2)c2cc(N3CCCCC3)c(F)cc2c1=O	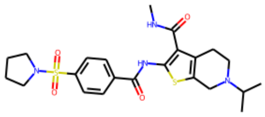	-	+	128.2
21	CN1C(=O)NC(C(C(=O)c2cccs2)C1(O)C(F)(F)F)c1ccc(O)cc1	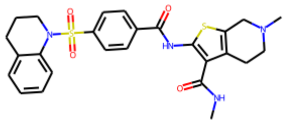	-	+	683.5
22	CCCN1CCc2c(C1)sc(NC(=O)c1ccc(cc1)S(=O)(=O)N(C)c1ccccc1)c2C(=O)NC	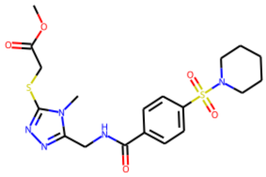	+	+	109.9
23	CCc1ccc(cc1)S(=O)(=O)c1cn(Cc2ccccc2)c2cc(N3CCCC3)c(F)cc2c1=O	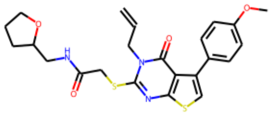	+	+	105.3

24	CNC(=O)c1c(NC(=O)c2ccc(cc2)S(=O)(=O)N2CCCC2)sc2CN(CCc12)C(C)C	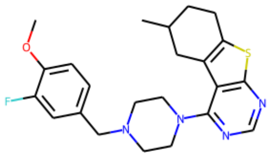	+	+	38.5
25	CN(C)c1ccc(CN(C2CCS(=O)(=O)C2)C(=O)c2cc3ccccc3oc2=O)cc1	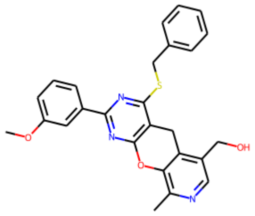	+	+	46.2
26	CC1CCN(CC1)c1cc2n(Cc3ccccc3)cc(c(=O)c2cc1F)S(=O)(=O)c1ccc(C)c1	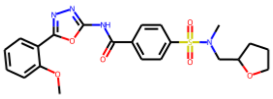	+	+	126.8
27	Cc1ccc(cc1S(=O)(=O)N1CCCCC1)C(=O)Nc1cccc2cnccc12	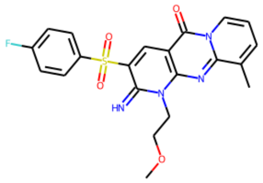	+	+	69.6
28	CC1(C)COc2ccc(NS(=O)(=O)c3ccc4CCCCc4c3)cc2N(CC=C)C1=O	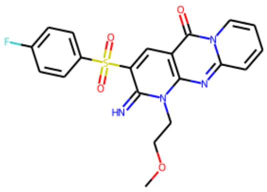	-	-	63.1
29	CNC(=O)c1c(NC(=O)c2ccc(cc2)S(=O)(=O)N2CCCc3ccccc23)sc2CN(C)CCc12	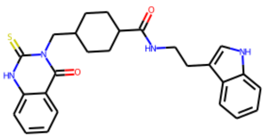	+	-	135.2
